# A Full-Image Deep Segmenter for CT Images in Breast Cancer Radiotherapy Treatment

**DOI:** 10.3389/fonc.2019.00677

**Published:** 2019-07-25

**Authors:** Jan Schreier, Francesca Attanasi, Hannu Laaksonen

**Affiliations:** Varian Medical Systems, Palo Alto, CA, United States

**Keywords:** machine learning, segmentation, breast, neural network, radiation therapy

## Abstract

Radiation therapy is one of the key cancer treatment options. To avoid adverse effects in the healthy tissue, the treatment plan needs to be based on accurate anatomical models of the patient. In this work, an automatic segmentation solution for both female breasts and the heart is constructed using deep learning. Our newly developed deep neural networks perform better than the current state-of-the-art neural networks while improving inference speed by an order of magnitude. While manual segmentation by clinicians takes around 20 min, our automatic segmentation takes less than a second with an average of 3 min manual correction time. Thus, our proposed solution can have a huge impact on the workload of clinical staff and on the standardization of care.

## 1. Introduction

For radiation therapy treatment planning, the anatomical structures need to be segmented from computed tomography or magnetic resonance imaging scans. The segmentation is currently done by a trained clinical expert and it takes, in the case of breast cancer, on average 34 min per patient and 6.5 min per organ-at-risk(OAR) (1). This generates cost for care providers and makes adaptive planning approaches, which use the anatomy of the day, not feasible in many cases.

In the case of breast cancer, for treatment planning purposes commonly both ipsi and contra lateral breasts and the heart are contoured. However, the delineation of the breasts is poorly defined in comparison to, e.g., the segmentation of the lungs or the heart. The reason for this is the diffuse boundary between the breast tissue and the surrounding fat tissue. Therefore, a high inter-observer variability exists. This can change the position of the border on average between 1.6 to 8.5 mm (2) and with a mean standard deviation of 5.7 mm (3). Additionally, a multi-institutional and multi-observer study reported a standard deviation of up to 60% in volumetric variation (4). Thus, the definition of the correct contour is inherently influenced by the person performing the contouring work. An automatic contouring algorithm could therefore accelerate the delineation process and improve consistency between observers. The automation of the breast segmentation has been achieved using atlas methods (5) or filter methods (6). However, through the advancement of deep learning in other fields of science, we can create more robust algorithms, which can adapt better to different anatomies.

### 1.1. Related Work

Atlas methods are successful in image segmentation of the brain (7, 8) and the breast (5). In atlas methods, the patient is registered to an atlas patient and the segmentations of the atlas patient are transformed to the patient coordinate space. One hindrance here is that for each segmentation a patient needs to be chosen from a patient library whose anatomy is similar. In addition to that, 5 to 10% of the volume still needs editing (5).

Another approach is the combination of locally adaptive filters in combination with heuristic rules (6). This has been done using a tunable Gabor filter yielding a robust and accurate segmentation on axial MR slices. One advantage of their method over deep learning is that it is independent of any training data. With deep learning, the network is generally only able to be applied to cases which are similar to the data it has seen whereby robustness cannot be ensured. In addition, machine learning approaches are always in need of a large number of training cases, whereas tunable filters are designed independent of the training cases. One advantage of our approach, however, is that the model architecture can be easily applied to different organs and different anatomical sites, provided the training data are available. Thus, the tedious work of defining heuristic rules and exploring filter options for each organ can be omitted.

Deep neural networks for segmentation typically use a structure similar to auto-encoders, in the sense that a dimension reduction is followed by a reconstruction network. Differences exist, however, in whether the spatial information is completely omitted such as in the anatomically constrained neural network (ACNN) (9), or if the spatial resolution is only reduced, as for example in the U-Net (10). The latter has been used for segmentation of CT images of pancreatic tumor (11) and liver (12). However, those approaches either use a 2D U-Net or are in need of another neural network on top of the U-Net. The skip connections from the downward path to the reconstruction path of the U-Net are an important improvement over convolutional neural networks (CNNs) as they help preserve more detailed spatial information for the reconstruction. Additionally, Drozdal et al. (13) propose the use of short skip connections, which improve the segmentation quality.

In a recent approach, the proposed network uses the shape of a U-Net but includes residual blocks both in the downward path and the reconstruction part (14). Additionally, a fully connected layer is constructed parallel to the lowest resolution level. The advantage is that through this approach the benefits from Ronneberger's U-Net and the Oktay's ACNN are combined. One downside however is that, due to the fully connected layer, the input size cannot be adapted during inference to the size of the CT image. Additionally, the inference is performed slice-wise. Even though this allows processing the image in full resolution, it deteriorates the inference speed compared to full-image processing. However, this particular model has a large capacity and can handle 21 organs with one inference.

The goal of this work is to improve the inference speed of a deep neural network for the segmentation of the organs needed for radiotherapy treatment planning, while maintaining state-of-the-art segmentation quality. We focus on the ipsi and contra-lateral breasts and the heart. The approach, here, is to replace the patch-wise or slice-wise processing by a full-image processing approach. The proposed network structure is a combination of the U-Net and the ResNet.

## 2. Materials and Methods

### 2.1. Model Architecture

We construct a fully convolutional neural network, which we refer to as BibNet in the following, due to its bib-like shape. For this network, the input and the output size is equivalent to the full image size, typically 256 × 256 × 128 pixels (xyz). Due to its fully convolutional structure, the network size can be adapted to the incoming input size. The network inherits its basic shape from the U-Net proposed by Ronneberger et al. (10). In addition to the U-Net architecture, connections on all resolution levels are added. These connections themselves process the image and are interconnected with layers of higher and lower resolution levels. Therefore, processing of features of different resolution scales becomes possible. Furthermore, the network is deeper with the concept of residual connections, which have proven to increase the performance of a neural network (15). In addition to that, in contrast to the U-Net proposed by Ronneberger et al. (10), we use transpose convolutions instead of upsampling followed by a convolutional layer.

**Figure 1:**
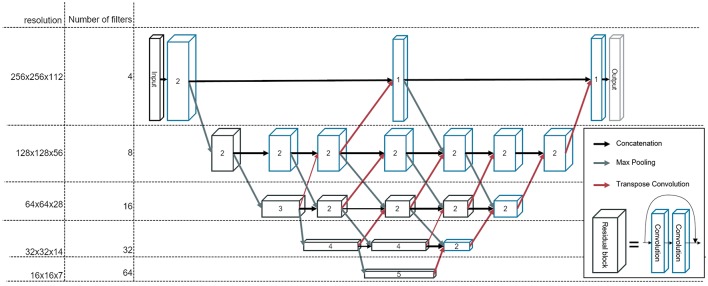
The architecture of our newly developed network, called BibNet. Here, the blue boxes represent convolutional blocks with each block comprising a dropout layer, a convolutional layer, an activation layer and a batch normalization layer. The black boxes represent residual blocks; each comprising two convolutional blocks and a skip connection. The number inside the box represent the number of convolutional or residual blocks which are appended. The arrows indicate the connections in between the convolutional and residual blocks: the black arrows are forwarding the output of the previous block to the input of the next block, whereas the gray arrows symbolize a strided convolution, which is performed to decrease the resolution of the output of the previous layer. Additionally, the red arrows indicate transposed convolutions, which are used to double the resolution in each dimension.

The network architecture is illustrated in Figure 1. The strided convolutions use a filter size of (2,2,2) and both the strided and the transpose convolutions have a stride factor of two in each dimension. In order to prevent bottlenecking in the lower resolution levels, the number of filters per layer is increased by a factor of two after each pooling. The filters themselves are convolutional filters with a shape of (3,3,3) with padding applied. Therefore, each dimension is treated equally and the image size is equivalent before and after the convolution.

The parameterized rectified linear unit (PReLU) is used as the activation function and the dropout rate is set to 0.5. The number of parameters for this network is ~7.5 million.

### 2.2. Data and Preprocessing

We use a data set consisting of 251 CT scans from female breast cancer patients with intact breasts in head-first supine position. The data set is split into a training, a validation and a test set, which are randomly selected. The training set contains 149 scans, the validation set 50 scans and the test set 52 scans. Additionally, a fourth data set is used for a robustness test. This data set stems from a hospital in North America and includes 64 patients. The position of the patients in the robustness set differ from those in the training set: The patients in the robustness set are imaged with one arm down, whereas the patients in the training set are imaged with both arms up.

The images and structure sets originate from three different European clinics. The contours are reviewed and missing structures added by experienced dosimetrists. As the voxel spacing differs depending on the clinic between 0.98 and 1.27 mm and the slice thickness between 2.5 and 3 mm, we resample the images to a common voxel spacing of 1.17 mm and a slice thickness of 3 mm. As a second step, we downsample the images by a factor of two in the axial slice to improve training speed and reduce the memory consumption. The downsampling is performed using trilinear interpolation on the CT scans and the corresponding structure sets, leading to a final voxel spacing of 2.34 mm in the axial slice and 3 mm slice thickness.

### 2.3. Computing Platform

As a high-level interface, a deep learning framework developed in-house by Varian is used. This is necessary to generate the 3D patches for training. It builds on top of Keras, which uses Tensorflow as a backend. Tensorflow itself then uses CUDA 9.0 and the corresponding cuDNN library.

### 2.4. Loss Function

The loss function or error function tries to measure how well the model performs with respect to this measure. The loss function can, therefore, be used to adjust the network to a desirable result.

For a pixel-wise output, such as image segmentations, a simple loss function is the binary categorical accuracy. It is defined as the percentage of the pixels which are correctly classified.

For medical segmentations, as large parts of the image belong to the background, the binary categorical accuracy can lead to false classification of the whole image to the background class. Therefore, a definition of the loss using the intersection and union of the segmentation with the ground truth is helpful. One loss function is the Sørensen-Dice coefficient defined as:

(1) $$\begin{array}{r} DSC=\frac{2|X\cap Y|}{|X|+|Y|} \end{array}$$

where *X* and *Y* are the sets of voxels classified to belong to one class through the algorithm and through the human expert respectively. This can be rewritten as:

(2) $$\begin{array}{r} DSC=\frac{2TP}{2TP+FP+FN} \end{array}$$

with *TP*: true positives, *FP*: false positives and *FN*: false negatives. The Dice loss can then be defined as 1 − *DSC*.

Another loss function is the Jaccard loss, which is based on the Jaccard index defined as (16):

(3) $$\begin{array}{r} r=\frac{|X\cap Y|}{|X\cup Y|} \end{array}$$

The Jaccard loss can then be defined as:

(4) $$\begin{array}{r} J=1-r \end{array}$$

During the training, the Jaccard loss is used as a loss function for the back-propagation. The Dice loss on the other hand is used during the hyper search for an initial scoring of the model. It is also used for evaluation after training the models.

### 2.5. Training

The networks are trained on a Nvidia Quadro P5000, a P4000 or a K80 until convergence. During training the patch size is reduced to 256 × 256 × 48 to be within the memory constraints. For the evaluation of this model, we increase the patch size to 256 × 256 × 128, such that the whole image can be processed at once. We train two different types of networks: single-organ and multi-organ networks. While the single-organ networks only segment one of the three organs at a time, the multi-organ network segment all three at once. The idea behind the latter is that basic image processing within the neural network is shared for the different organs and, thus, the network can generate different structures at the same time.

The patches for training are sampled using a combination of an entropy sampler, two shell samplers and a mask sampler. The sampling strategy is vital for the training of the 3-level U-Net, as the image is processed in small patches. The entropy sampler chooses random samples with an entropy higher than a certain threshold. This tries to avoid patches taken from plain surfaces, such as the air surrounding the body. The shell sampler generates patches, which are at most 20 or 40 mm away from the surface of a ground truth structure. The mask sampler, on the other side, restricts patches to be taken only if they intersect with the ground truth. The choice of the sampler might be less relevant for the BibNet as here larger parts of the image are processed at the same time.

As an optimizer, the Nesterov Adam optimizer (17) is used. This optimizer combines the adaptive momentum update (Adam) (18) with the idea from Nesterov (19) to apply the gradient only after the momentum update has been done. It can improve the convergence rate and minimize the loss more effectively than Adam. The learning rate is initially set to 10^−2^ and then linearly decreased during the training to a minimum of 10^−5^.

To improve the performance of the model, we initialize eight networks with random weights. We train these networks and drop half of the networks at 20, 40, and 80 epochs. The networks dropped are the ones with the worst performance on the validation set. At the end, we choose the best performing one on the validation set and train it until the validation loss does not decrease further.

### 2.6. U-Net

In order to compare the newly built network, the U-Net-like architecture proposed by Hänsch et al. (20) is reproduced. This architecture is a 3-level UNet with two convolution layers and a max-pooling layer per level in the encoder part and two convolution and one upsampling layer per level in the decoding part. The filters are (3,3,3) convolutional filters without padding. In the first level, there are 32 filters in each of the convolutional layers; in the second, 64 in the first and 128 in the second convolutional layer. In the lowest layer, there are 256 filters in each of the two convolutional layers. In the upward path, there are 128 and 64 filters per convolutional layer for the lower and upper level respectively. The training is performed on the same data set and the model is trained until convergence.

With this architecture, the 3D images are processed in patches, meaning that the network is applied to a subset of the actual image, which then outputs an even smaller subset. Afterwards, the receptive field of the network is shifted and the next output patch generated.

### 2.7. Evaluation Metrics

To be able to compare our model, an appropriate metric needs to be chosen. The Dice score can give an indication for the quality of the model. It is also useful for comparison to the works done by other researchers. However, it usually achieves higher scores for bigger organs, as the main problems occur on the boundary and gives, therefore, a less meaningful measure when comparing the quality of the segmentation between different organs.

Thus, a more informative metric is the average surface distance between the segmentation and the ground truth. Here for each point of the surface of the segmentation, the distance *r* to the closest point on the surface of the ground truth is measured. Then, for each organ the root-mean-square (RMS) value of these distances is calculated:

(5) $$\begin{array}{r} \text{rms surface distance}=\sqrt{\underset{x_{i}\in P}{\sum }{\left(\underset{q\in G}{\min}|x_{i}-q|\right)}^{2}} \end{array}$$

with *P* being the set of surface points of the prediction and *G* the set of surface points of the ground truth.

Another common metrics is the Hausdorff distance, which is defined as (21):

(6) $$\begin{array}{r} D_{H}\left(P,G\right)=\max\left(\underset{x\in P}{\sup}\underset{q\in G}{\inf}d\left(x,q\right),\underset{q\in G}{\sup}\underset{x\in P}{\inf}d\left(x,q\right)\right) \end{array}$$

with *d* being the Euclidean distance. This distance measures the maximum distance between the two surfaces, whereas the rms surface distance measures an average of the distance.

For reporting the score of a model, we report the median value of the scores per organ and model. The median is chosen as even one failing segmentation can deteriorate the mean score significantly. The mean values are however reported for the comparison with the results from Men et al. (22).

### 2.8. Statistical Methods

For comparing the different models, a paired two-tailed *T*-test for the means is performed. The significance threshold is set to 5%. However, as we have three different models, a multi-comparison problem exists. Thus, the α-value is adjusted using the Bonferroni correction to α = 0.05/3 = 0.0167.

### 2.9. Inference Time

The inference time is measured on a machine with the Nvidia P5000 graphics card, two Intel Xeon E5-2640 v4 at 2.40 GHz and 64 GB memory. Here, only the actual inference time is taken into account, not including, for instance, the loading of modules such as Tensorflow and initializing the Tensorflow graph. For the timing, Tensorflow version 1.6.0 is used.

In addition to this, for more reproducible results, a NC6_v2 virtual machine(Nvidia Quadro P100 graphics card, 6 Intel Xeon E5-2690 v3 2.60GHz processor cores and 128 GB memory) is instantiated on Azure with the pre-configured “Ubuntu Data Science” image. This image contains Cuda 9.0 and the Nvidia Docker runtime. On this machine, the Tensorflow serving docker image with version 1.12.0 for GPU and CPU is run and an image of size 256 × 256 × 128 (xyz) inferred using gRPC through the local host. In order to reduce in memory transfer times, the input image is in uint8 and gzip as compression is applied. The timing is taken for 11 consecutive prediction requests, where the channel is already created. The first of each timings is discarded to ensure that the model is already loaded by tensorflow serving.

### 2.10. Required Correction Time

Even though different metrics are able to show the similarity between human-created structures and structures stemming from deep learning models, these differences need to be translated into the impact on the clinical work flow. To address this, two experienced dosimetrists and two radiation oncology specialists are asked to correct structures stemming from the single-organ bibnet with the goal of producing clinically acceptable segmentation according to the RTOG consensus contouring guideline (23). For this exercise, 7 patients are used, which were not included in the training: one patients from each of the three clinics of the test set and four patients from a clinic in North America. The output of the models is resampled to the resolution of the CT images using trilinear interpolation. Furthermore, Gaussian smoothening is applied to avoid aliasing effects of the resampling and the largest connected component taken. The correction is done using Eclipse Treatment Planning Software (Varian Medical Systems, Palo Alto), which is familiar to all participants. The time needed for the correction for each organ is measured.

## 3. Results

The inference time on the Azure machine using Tensorflow Serving on GPU for a full image with a resolution of 256 × 256 × 128 (xyz) is 1.13(1) s for the multi-organ BibNet, 3.38(5) s for the single-organ BibNet and 12.4(1) s for the U-Net. On CPU, the inference times are 16.30(3) s for the multi-organ BibNet, 48.9(1) s for the single-organ BibNet and 253(2) s for the U-Net.

The inference time on the local machine for a full image with a resolution of 256 × 256 × 128 (xyz) on GPU is 10.68 s for the U-Net, 1.92 s for the single-organ BibNet and 0.64 s for the multi-organ BibNet. On CPU, the inference times are 19 min 2 s for the U-Net, 7 min 8 s for the single-organ BibNet and 2 min 18 s for the multi-organ BibNet.

The median Dice scores for the three different organs for the patients in the test set as well as the inference times for the different models using Tensorflow serving can be seen in Table 1.

**Table 1:** The median Dice scores for the patients in the test set, and inference times with standard deviation in parenthesis using Tensorflow Serving for the different models.

	**Dice score**			**Inference time in s**	
**Model**	**Left breast**	**Right breast**	**Heart**	**GPU**	**CPU**
BibNet single-organ	**0.924**	0.929	**0.951**	3.38 (5)	48.9 (1)
BibNet multi-organ	**0.924**	**0.935**	**0.949**	**1.13 (1)**	**16.30 (3)**
3-level U-Net	0.910	0.911	0.935	12.4 (1)	253 (2)

*The best value for each category is marked in bold*.

**Figure 2:**
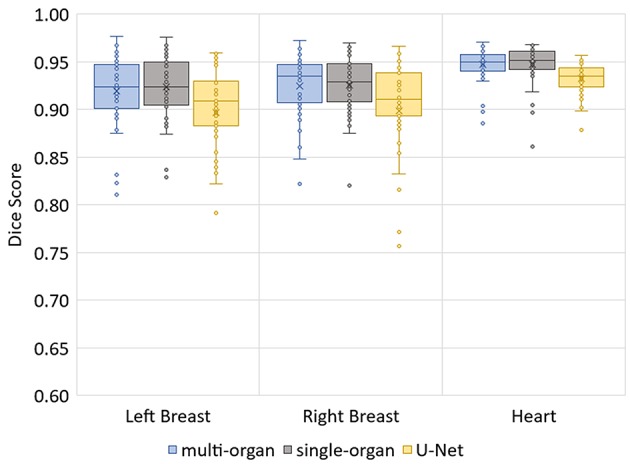
Box-plot of the Dice scores of the three networks on the test set.

**Figure 3:**
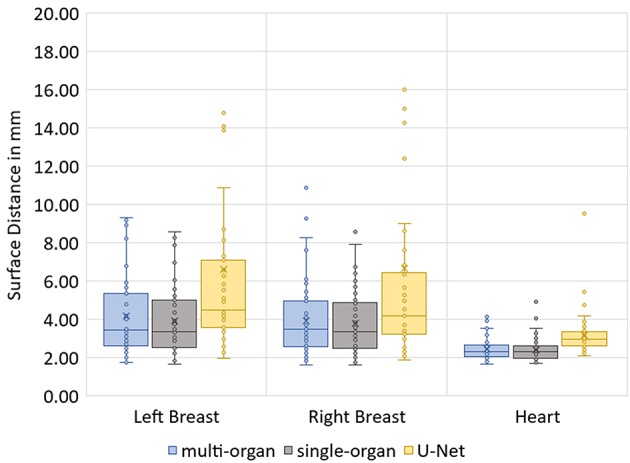
Box-plot of the RMS surface distances of the three networks on the test set.

The median Hausdorff distance and median RMS surface distance for the three different organs for the patients in the test set can be seen in Table 2. The median values cannot characterize either the inter-subject variability of the inference quality or the robustness of the network. Therefore, box-plots for the Dice scores are shown in Figure 2 and for the RMS surface distance in Figure 3. In the latter, for the U-Net two outliers at 26.8 and 41.6 mm on the left breast and on the right breast two outliers at 34.6 and 53.4 mm are not visible. Additionally, one patient, which was imaged with contrast agent, was removed from the analysis for the heart, since here the U-Net was unable to produce a structure.

**Table 2:** The median Hausdorff and RMS surface distances in millimeter for the patients in the test set.

	**Hausdorff distance**			**Surface distance**		
**Model**	**Left breast**	**Right breast**	**Heart**	**Left breast**	**Right breast**	**Heart**
BibNet single-organ	**20.6**	**20.0**	**8.5**	**3.36**	**3.36**	**2.31**
BibNet multi-organ	21.3	20.8	**8.5**	3.47	3.71	2.34
3-level U-Net	26.4	25.6	11.4	4.49	4.20	2.98

*The best value for each category is marked in bold*.

The p-values of the two-tailed paired *t*-test for Dice scores and surface distance are shown in Table 3.

**Table 3:** The p-values (in decimals) of the two-tailed paired *t*-test for Dice scores and surface distance in comparison of the different models.

	**Dice score**			**Surface distance**		
**Model**	**Left breast**	**Right breast**	**Heart**	**Left breast**	**Right breast**	**Heart**
BibNet single-organ vs multi-organ	0.0096	0.34	0.89	0.042	0.132	0.796
BibNet multi-organ vs 3-level U-Net	0.0078	0.0061	< 0.0001	0.0040	0.022	< 0.0001
BibNet single-organ vs 3-level U-Net	0.0043	0.0057	< 0.0001	0.0020	0.016	< 0.0001

*The scores, which are statistically significant, are marked with a gray background*.

The median dice score, surface distance and Hausdorff distance for the three models applied to the robustness data set is shown in Table 4.

**Table 4:** The median dice score, surface distance (in mm) and Hausdorff distance (in mm) for the three models applied to the 64 patients of the robustness data set.

	**Dice score**			**Surface distance**			**Hausdorff distance**		
**Model**	**Left breast**	**Right breast**	**Heart**	**Left breast**	**Right breast**	**Heart**	**Left breast**	**Right breast**	**Heart**
BibNet single-organ	**0.935**	**0.938**	0.966	**3.69**	**3.58**	1.86	**21.2**	**22.2**	7.2
BibNet multi-organ	0.929	0.928	**0.967**	4.14	4.01	**1.84**	24.4	25.2	**6.3**
3-level U-Net	0.900	0.917	0.942	5.99	5.74	2.96	36.4	41.3	12.8

*The best performing model in each category is marked with bold letters*.

**Figure 4:**
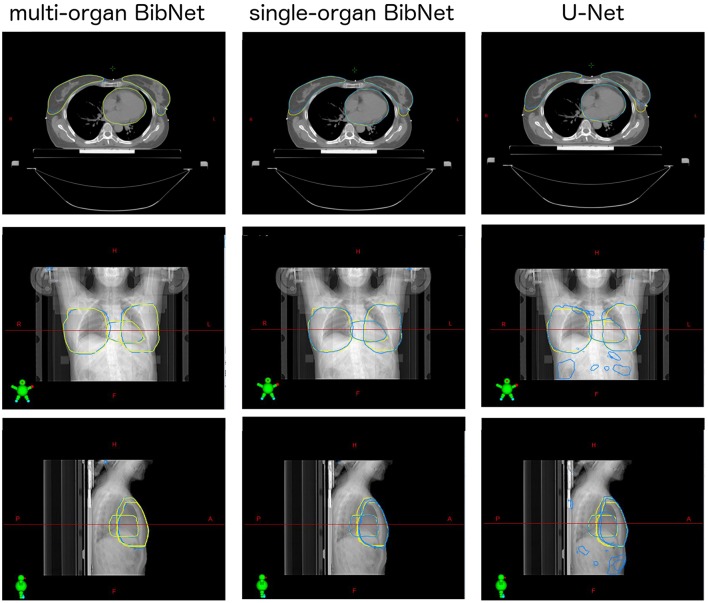
Axial slices of one breast cancer patient from the test set as well as frontal and sagittal topograms. The structures stem either from a clinical expert (yellow) or from one of the three models (blue). For the axial slices, Gaussian smoothening is applied and the largest connected component taken. The topograms display the raw output of the models without post processing.

For one test patient, axial slices with the segmentations done by the three networks and by a clinical expert as well as sagittal and frontal topograms are shown in Figure 4. For the axial slices, the largest connected component is taken and a Gaussian smoothening applied. The topograms, however, show the raw output of the networks.

**Figure 5:**
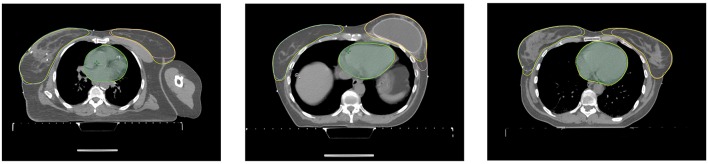
Axial slices of three patients from the correction time test. Each axial slice has the structures stemming from the model and the corrected structures (yellow) from the dosimetrist.

The correction time needed for each of the organs of the seven patients by the two dosimetrists and two radiation oncology specialists are shown in Table 5. The average correction time for the heart is 33s, for the left breast 101s and for the right breast 97s. For three of the patients, the generated structures as well as the corrected structures from one dosimetrist are shown in Figure 5.

**Table 5:** The average correction time and standard deviation in minutes needed for each of the organs of the seven patients by the two dosimetrists and two radiation oncology specialists.

**Corrector**	**Left breast**	**Right breast**	**Heart**	**Per patient**
Dosimetrist 1	1.5 ± 0.6	1.0 ± 0.2	0.2 ± 0.1	2.7 ± 0.7
Dosimetrist 2	1.8 ± 0.6	1.1 ± 0.2	0.3 ± 0.1	3.2 ± 0.5
Radiation oncology specialist 1	1.4 ± 0.5	1.7 ± 0.9	0.8 ± 0.2	3.9 ± 1.2
Radiation oncology specialist 2	2.1 ± 1.5	2.7 ± 0.9	0.8 ± 0.2	5.6 ± 1.1
Average	1.7 ± 1.0	1.6 ± 0.9	0.5 ± 0.4	3.9 ± 1.4

*The original structures stem from the single-organ bibnet and are corrected to be clinically applicable according to the RTOG contouring guidelines. Four patients stem from one North American clinic and one patient from each of the three different European clinics*.

## 4. Discussion

The main observation is the inference time: the newly constructed multi-organ BibNet performs the inference 10 times faster than the U-Net on GPU and 15 times faster on CPU. Through the full-image architecture of the BibNet, the inference needs to be performed only once, whereas the U-Net needs to perform the inference several times as it processes the image in patches. The single-organ BibNet needs to perform the inference for each organ separately and is therefore three times slower than the multi-organ BibNet.

The time difference between the local implementation using Tensorflow 1.6.0 and Tensorflow serving 1.12.0 stem from two different causes: Firstly, using Tensorflow serving creates an overhead compared to the plain inference time measured with Tensorflow as the gRPC request needs to be sent. This effect is reduced through the usage of uint8 and compression, which both lead to reduced transfer times. Secondly, the inference time on CPU was improved between Tensorflow 1.6 and 1.12, whereas the inference time on GPU is nearly unchanged. Thus, the local inference is faster on GPU as the transfer times are larger than the improvement on GPU speed, whereas the opposite holds true for the CPU.

The average correction time combined for the three structures created by the single-organ bibnet is 4 min. This stands in comparison to the average manual contouring time per organ-at-risk of 6.5 min in clinical practice (1). Therefore, the proposed deep neural network is able to save ~15.5 min per patient in the clinical work flow if integrated seamlessly.

The Dice scores, surface distances and Hausdorff distances show that the segmentation quality is better for the two BibNet models than for the 3-level U-Net proposed by Hänsch et al. (20). In addition to that, the BibNet seems to be more robust than the U-Net as several outliers occur with the latter.

The differences between the single-organ and multi-organ BibNet are not statistically significant (p-value < 1.67%) except for the dice score of the left breast. Here, the mean dice score is higher for the single-organ BibNet.

The robustness test shows that both BibNets are able to perform equally good on patients from a different clinic as from the training clinics. This is surprising as the different patient position affects the anatomy of the contralateral breast and as several of the patients have breast implants. Both the Hausdorff distance and the surface distance show that the U-Net is performing worse for both breasts on the robustness data set. This indicates that the BibNet is more robust than the U-Net, which might be due to the greater receptive field of the BibNet and the smaller input size of the U-Net of 68 × 68 × 64 (xyz). For the BibNet, every neuron in the last layer of the encoding part has a receptive field of 484 × 484 × 484 (xyz) and can, thus, see the full input image, whereas the input of the U-Net is fixed to a smaller patch. Therefore, a larger context is available to the BibNet compared to the U-Net.

The performance of the heart segmentation of all three models is equivalent between the test patients from the training clinics and the robustness test clinic. This might be due to the reduced dependency of the heart structure on the patient position.

When inspecting the raw output of the three models on the topograms, it can be seen that the breast structure is contoured too far superior in the U-Net compared to the expert's contour. The two BibNet networks are able to follow the shape better in the superior part. However, the two networks are unable to produce the sharp cut on the upper part of the breast. This might be because there are no clear anatomical landmarks where to cut and the sharp cut is not necessarily following any actual anatomical structure.

The U-Net yields several false positive clusters, whereas the two BibNet models each only produce one small false positive cluster on the superior end of the image. This might be due to the smaller receptive field and input size of the U-Net. Therefore, the context for the inference is missing, leading to false positives, for example, in the fat tissue of the arm or of the belly.

The small false positive cluster produced by each of the two BibNets can be easily mitigated through maintaining only the largest connected component. This approach, however, is prone to failure for the U-Net, since here the clusters are of similar size as the actual organ and are close to the actual structure. Thus, it is likely that these clusters could connect to the actual segmentation. In the presented topograms in Figure 4, the false positive clusters of the heart are one example of these large clusters.

Recently, Men et al. used 2D networks in breast segmentation in slice-by-slice manner (22). In their approach, several fully convolutional networks were trained on 800 breast cases. The networks achieve an average Dice score of 0.91 for the breast on their test set. In comparison, the average Dice score for the single-organ and multi-organ BibNet is 0.92 for both networks on the left breast and 0.93 on the right breast. Hence, it shows that our networks seem to perform slightly better on average. One main limitation with this comparison is that no common test set was applied. Therefore, some anatomies might be more difficult, and the ground truths might be of varying quality and consistency. In addition to that, the data sets seem to be differing in the sense that different contouring practices have been applied. For instance in our data set, the breast is contoured more laterally.

In the literature, high Dice scores are reported using a classical method of locally tunable Gabor filter (6). The comparability between their study and the present work is however deteriorated through the different imaging modality and patient position used. In their work, the MRI images were taken in prone position using a specialized breast coil. Therefore, the breasts are not laying on top of the patient, which makes the border between the fat tissue and the actual breast on the superior and inferior boarder easier to distinguish. Also, the resolution is better in the MRI images. The processing time on CPU per full 3D image is 4.1 min in their approach compared to 16 s in our approach using the multi-organ BibNet. Therefore, the multi-organ BibNet is faster by a factor of 15. This reduces the interruption in the clinical workflow when using automated segmentation.

In conclusion, we introduced a novel neural network architecture, BibNet, that combines the basic shape of a U-Net with added multi-resolution level processing and residual connections. The new architecture together with the change from a patch-wise processing to a full-image processing is able to increase the inference speed by an order of magnitude, while also improving the segmentation quality. As the inference speed is of the order of a second and the correction time around 3 min, the automatic segmentation has the potential of not only simplifying the workflow for treatment planning, but might also open possibilities for adaptive radiation therapy by allowing to change the radiation plan to the anatomy of the day.

## Data Availability

The datasets for this manuscript are not publicly available because they are Varian proprietary data. Requests to access the datasets should be directed to JS, jan.schreier@varian.com.

## Author Contributions

JS initiated the work, designed the neural network, ran training, and evaluated the results. FA was responsible for data collection and curation. HL supervised the work presented. All authors contributed to the manuscript.

### Conflict of Interest Statement

The authors are employed by Varian Medical Systems, Palo Alto, CA, which funded the research of this work.
